# GPT-4-based AI agents—the new expert system for detection of antimicrobial resistance mechanisms?

**DOI:** 10.1128/jcm.00689-24

**Published:** 2024-10-17

**Authors:** Christian G. Giske, Michelle Bressan, Farah Fiechter, Vladimira Hinic, Stefano Mancini, Oliver Nolte, Adrian Egli

**Affiliations:** 1 Division of Clinical Microbiology, Department of Laboratory Medicine, Karolinska Institutet, Stockholm, Sweden; 2 Department of Clinical Microbiology, Karolinska University Hospital, Solna, Sweden; 3 Institute of Medical Microbiology, University of Zurich, Zurich, Switzerland; Johns Hopkins University, Baltimore, Maryland, USA

**Keywords:** EUCAST, antimicrobial resistance, diagnostics, screening, artificial intelligence, ChatGPT, GPT-4, AI agent, Kirby-Bauer method, inhibition zones, large language model, multi-modal AI

## Abstract

The European Committee on Antimicrobial Susceptibility Testing (EUCAST) recommends two steps for detecting beta-lactamases in Gram-negative bacteria. Screening for potential extended-spectrum beta-lactamase (ESBL), plasmid-mediated AmpC beta-lactamase, or carbapenemase production is confirmed. We aimed to validate generative pre-trained transformer (GPT)-4 and GPT-agent for pre-classification of disk diffusion to indicate potential beta-lactamases. We assigned 225 Gram-negative isolates based on phenotypic resistances against beta-lactam antibiotics and additional tests to one or more resistance mechanisms as follows: “none,” “ESBL,” “AmpC,” or “carbapenemase.” Next, we customized a GPT-agent with EUCAST guidelines and breakpoint table (v13.1). We compared routine diagnostics (reference) to those of (i) EUCAST-GPT-expert, (ii) microbiologists, and (iii) non-customized GPT-4. We determined sensitivities and specificities to flag suspect resistances. Three microbiologists showed concordance in 814/862 (94.4%) phenotypic categories and were used in median eight words (interquartile range [IQR] 4–11) for reasoning. Median sensitivity/specificity for ESBL, AmpC, and carbapenemase were 98%/99.1%, 96.8%/97.1%, and 95.5%/98.5%, respectively. Three prompts of EUCAST-GPT-expert showed concordance in 706/862 (81.9%) categories but were used in median 158 words (IQR 140–174) for reasoning. Sensitivity/specificity for ESBL, AmpC, and carbapenemase prediction were 95.4%/69.23%, 96.9%/86.3%, and 100%/98.8%, respectively. Non-customized GPT-4 could interpret 169/862 (19.6%) categories, and 137/169 (81.1%) agreed with routine diagnostics. Non-customized GPT-4 was used in median 85 words (IQR 72–105) for reasoning. Microbiologists showed higher concordance and shorter argumentations compared to GPT-agents. Humans showed higher specificities compared to GPT-agents. GPT-agent’s unspecific flagging of ESBL and AmpC potentially results in additional testing, diagnostic delays, and higher costs. GPT-4 is not approved by regulatory bodies, but validation of large language models is needed.

**IMPORTANCE:**

The study titled "GPT-4-based AI agents—the new expert system for detection of antimicrobial resistance mechanisms?" is critically important as it explores the integration of advanced artificial intelligence (AI) technologies, like generative pre-trained transformer (GPT)-4, into the field of laboratory medicine, specifically in the diagnostics of antimicrobial resistance (AMR). With the growing challenge of AMR, there is a pressing need for innovative solutions that can enhance diagnostic accuracy and efficiency. This research assesses the capability of AI to support the existing two-step confirmatory process recommended by the European Committee on Antimicrobial Susceptibility Testing for detecting beta-lactamases in Gram-negative bacteria. By potentially speeding up and improving the precision of initial screenings, AI could reduce the time to appropriate treatment interventions. Furthermore, this study is vital for validating the reliability and safety of AI tools in clinical settings, ensuring they meet stringent regulatory standards before they can be broadly implemented. This could herald a significant shift in how laboratory diagnostics are performed, ultimately leading to better patient outcomes.

## INTRODUCTION

As the healthcare sector grapples with the escalating challenge of antimicrobial resistance (AMR), the need for advanced diagnostic methods increases (1). For beta-lactamases in Gram-negative bacteria, this is usually a two-step process (2): first, based on screening breakpoints for minimal inhibitory concentrations (MICs) or inhibition zone diameters, suspected isolates with potential extended-spectrum beta-lactamase (ESBL), plasmid-mediated AmpC, and carbapenemase production are flagged. Depending on the geographical or regional conditions, laboratories may confirm the resistance mechanism with additional tests, e.g., combination disk testing, lateral flow assays for certain enzymes, or molecular assays for specific resistance genes (3).

Kirby–Bauer disk diffusion is a commonly used method for determining bacterial susceptibility and exemplifies the complexities of microbiological diagnostics (4, 5). This is particularly true for detection of extended-spectrum beta-lactamases (ESBLs), AmpC beta-lactamases, and carbapenemases. Precise interpretation within this framework is vital, yet challenged by technical and human variability, and there is necessity for continual expert knowledge updating. Reproducibility in reading and interpretation of disk diffusion have been reported to be variable (6–8).

In this context, the potential integration of AI technologies, such as generative models like generative pre-trained transformer (GPT)−4, customized GPT-agents (OpenAI), or other large language models (LLMs) into laboratory medicine is an area of growing interest (9). However, it is crucial to note that AI tools are not routinely established due to the current absence of compliance with *In Vitro* Diagnostic Regulation (IVDR) and Food and Drug Administration (FDA) regulations (10). This regulatory gap underscores the importance of validation to ensure their reliability, accuracy, and safety in clinical diagnostics.

Our study aimed to contribute to this validation process utilizing GPT-4 and a customized GPT-agent to interpret test results. We aimed to understand how such AI tools can be calibrated and utilized within the stringent frameworks of laboratory medicine. The study provides an opportunity to gather valuable insights into the integration of AI in diagnostics, for future validation and regulatory approval. This is a critical step in ensuring that AI tools can be safely and effectively used to enhance patient care in clinical laboratories (9, 11, 12).

## MATERIALS AND METHODS

### Study design and sample collection

We conducted a retrospective study involving 225 Gram-negative isolates originating from various clinical materials, including urine samples, blood cultures, and wound swabs, that were examined during routine diagnostics at the Institute of Medical Microbiology, University of Zurich, between May and November 2023. The isolates were retrospectively assigned to the study by a biomedical analyst, who was not otherwise involved in the study. Species identification was obtained with matrix-assisted laser desorption/ionization time-of-flight mass spectrometry (Bruker, Bremen, Germany). Laboratory processes are International Organization for Standardization/International Electrotechnical Commission (ISO/IEC) accredited. We randomly included four *Acinetobacter baumannii*, three *Citrobacter freundii*, two *Citrobacter koseri*, 13 *Enterobacter cloacae* complex, 132 *Escherichia coli*, five *Klebsiella aerogenes*, five *Klebsiella oxytoca* complex, 40 *Klebsiella pneumoniae*, three *Morganella morganii*, 10 *Proteus mirabilis*, one *Proteus vulgaris*, one *Pseudomonas aeruginosa*, and six *Serratia marcescens*.

The isolates were subjected to antimicrobial susceptibility testing with disk diffusion according to European Committee on Antimicrobial Susceptibility Testing (EUCAST) guidelines (13) using antibiotic discs purchased from i2a (Perols Cedex, France) and Mueller–Hinton agar plates (BD, Franklin Lakes, NJ). The SIRweb/SIRscan system (i2a, Montpellier, France) was used to measure the inhibition zone diameters (8). The detection of ESBL, AmpC, and carbapenemase production was based on the EUCAST guidelines for the detection of resistance mechanisms and included a two-step process consisting of an initial screening with the automated SIRweb expert system indicating zone diameters of cefoxitin, cefpodoxime, ceftazidime, ertapenem, or meropenem below screening breakpoints as well as manual checking for synergy phenomena. A positive screening was followed by confirmatory tests including combination disk testing according to EUCAST guidelines (3) and lateral flow assays for CTX-M beta-lactamase, NDM, IMP, VIM, KPC, or OXA-48-like carbapenemase production (NG Biotech, Guipry, France). Based on the results, each isolate was assigned one or more of the following four phenotypic resistance categories: “none” (*n* = 75), “ESBL” (*n* = 111), “AmpC” (*n* = 32), and “carbapenemase” (*n* = 23). “None” did not occur in combinations, as this indicated none of the other resistance mechanisms. A total of 4.2% of phenotypic resistance categories (38/900), corresponding to 10.2% of all isolates (23/225), had to be excluded. The main reasons were unclear, non-resulting interpretation by GPT-4 (*n* = 9), or a poor image quality (*n* = 7). We also excluded an output if there was a failure in one of the technical replicates as it would not have been possible to calculate the concordance between the triplicates (the customized GPT was asked independently three times for the reproducibility of the answer, like the three human experts). Also, we did not re-prompt or transform the image into another format, as we aimed for a consistent and comparable process for all images. Another reason for exclusion was due to a none-valid interpretation guideline, e.g., assessing AmpC in *Acinetobacter baumannii* and *Pseudomonas aeruginosa* (*n* = 5). We finally included a total of 862 valid category phenotypes for subsequent analysis. All data can be downloaded at DRYAD with the following link: DOI: 10.5061/dryad.5dv41nsfj.

### Development and application of the customized GPT-agent

**Fig 1:**
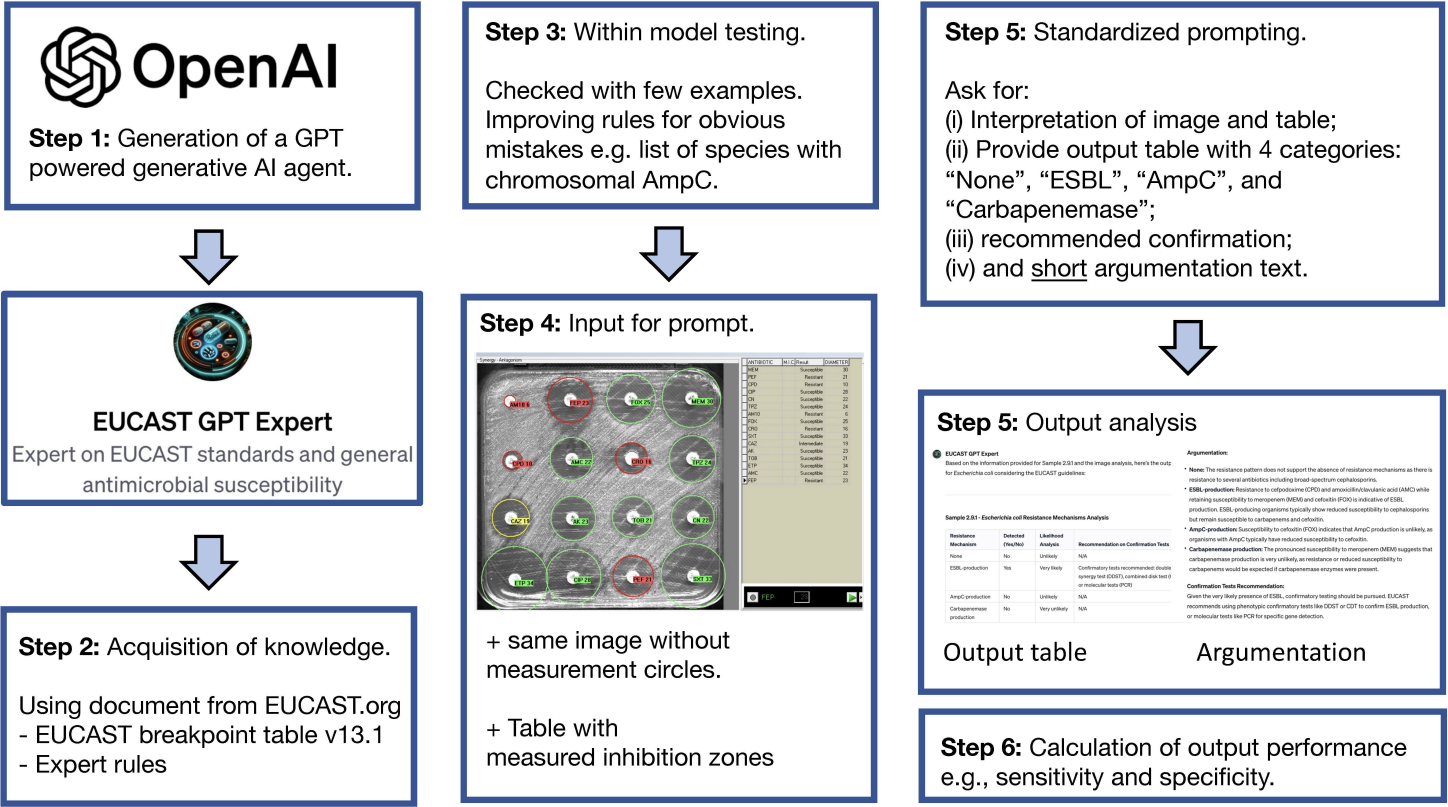
Workflow for validation of GPT-4 based generative AI-agent.

We generated a customized GPT-agent named “EUCAST-GPT-expert” using the commercial version of ChatGPT (OpenAI), which is a non-trainable LLM that can be customized to perform a specific task by providing documents containing further information about a particular topic. The specific GPT-4 version used was dated 6 November 2023 to assist in the interpretation of EUCAST antimicrobial susceptibility testing. The GPT-agent was customized and applied according to the following steps (Fig. 1; Fig. S1):

1. *Knowledge Acquisition*. The GPT-agent was customized by uploading with the following documents: latest breakpoint tables (v13.1 (14), EUCAST guidelines for detection of resistance mechanisms and specific resistances of clinical and/or epidemiological importance (v2, July 2017 [3]), and EUCAST expert rules (15, 16). This knowledge base was intended to enable the customized GPT-agent to understand and apply the EUCAST guidelines accurately.
2. *Model Refinement*. Preliminary testing was performed with some examples of disk diffusion images and a prototype prompt to determine the customized GPT-agent’s interpretative capabilities. As a result, the wording of the preliminary prompt was adjusted to ensure the GPT-agent did not repeat identifiable errors, such as miss-listing species known to have chromosomal AmpC with common de-repression, e.g., *Citrobacter freundii*, *Enterobacter cloacae*, *Klebsiella aerogenes*, etc., or providing awareness of intrinsic resistances such as ampicillin in *Klebsiella* spp.
3. *Input Preparation*. For each isolate, the input comprised images of disk diffusion plates from SIRscan. Accompanying these images was a table detailing the measured inhibition zones for each antibiotic (Fig. S2 and S3; Table S1).
4. *Standardized Prompting*. The GPT-agent was prompted in a structured manner to interpret the provided data (see below for exact prompt). The same prompt was also used for the non-customized GPT-4 and provided to human experts who are all board-approved microbiologists with at least 5 years of experience in routine diagnostics at the time. Each group was tasked to assess the isolates for the presence of the four resistance categories and to elaborate on its reasoning in a brief argumentative text including a recommendation on further confirmation tests for suspected resistance mechanisms.
5. *Output Analysis*. The analysis included a detailed output table containing the resistance categories and a section where the GPT-agent provided its argumentation for each categorization. For each bacterial isolate, the agent was prompted to analyse images and generate an output table identifying the suspected resistance mechanisms. The agent was instructed to force a binary (yes/no) decision on the presence of resistance mechanisms and to assess the likelihood of each identified mechanism.

### Standardized prompting procedure

For each sample, the identical queries were used. Example: “Sample 6.70.1. *Escherichia coli*—Make an output table. In that output table, identify the resistance mechanisms you have detected from the analysis of the provided images and information: (i) None, (ii) ESBL-production, (iii) AmpC-production, or (iv) Carbapenemase production. Make a specific call with yes/no answers—force yourself to provide an answer. Add into this table a likelihood analysis for each resistance mechanism: (i) very likely, (ii) likely, (iii) unlikely, (iv) very unlikely. For samples with likely or very likely results, make a recommendation on the potential confirmation tests, which should be used according to EUCAST. In the title of each table, mention the sample ID and the bacterial species. Provide a short argumentation for each resistance phenotype (none, ESBL, AmpC, and carbapenemase) based on the measurement and image analysis.

### Output analysis and argumentation

The output table from the EUCAST-GPT-expert included sample identification and resistance mechanism detection, with a likelihood analysis (Table S2). The GPT-agent was also required to provide a short argumentation for each decision, drawing on the measured inhibition zones and image analysis. This approach aimed to mimic the reasoning process of human experts.

### Benchmarking and validation

The outputs of all groups, GPT-4, GPT-agent, and three medical microbiologists were compared against the previously reported results in routine diagnostics (reference standard). All three microbiologists were board examed and had at least 5 years of working experience in routine diagnostics. As there was some variability in the interpretation of the results, we showed the median outputs of the three microbiological experts and performed three independent prompting rounds for the EUCAST-GPT-expert, where also the median outputs were used. The output of the non-customized GPT-4 was poor, and therefore, we did not repeat the prompting. We calculated concordance rates for categories identified by human experts and the EUCAST-GPT-expert. We recorded the median number of words used for reasoning.

### Statistical analysis

Descriptive statistics were used to summarize the performance of the human experts, the EUCAST-GPT-expert, and non-customized GPT-4. Sensitivity and specificity were compared. The median sensitivities and specificities for human readers were calculated with interquartile ranges (https://www.medcalc.org) and compared to evaluate the diagnostic accuracy of the AI models.

## RESULTS

The customized GPT-agent (“EUCAST-GPT-expert”), equipped with EUCAST guidelines and expert rules, analyzed 862 phenotypes from 225 Gram-negative bacterial isolates. When compared to the reference standard, the GPT-agent demonstrated a median sensitivity of 95.4% for ESBL detection, 96.9% for AmpC, and 100% for carbapenemase detection. Specificity, however, varied, with 69.2% for ESBL, 86.3% for AmpC, and 98.8% for carbapenemases, indicating a propensity for over-flagging potential resistances, particularly for ESBL and AmpC (Table 1). Of note, when the GPT-agent was asked to re-analyze and carefully consider the knowledge and rules, then the output was often adapted and corrected. For this analysis, we have, however, used only the first reply of the GPT-agent.

**TABLE 1:** Sensitivity and specificity of human experts and the customized EUCAST-GPT-expert

	Human experts^*a*^	EUCAST-GPT-expert^*b*^
ESBL		
Sensitivity	98.0% (91.8–100)	95.4% (94.5–96.3)
Specificity	99.1% (97.1–100)	69.2% (63.8–85.7)
AmpC		
Sensitivity	96.8% (93.3–100)	96.9% (87.5–96.9)
Specificity	97.1% (95.9–97.7)	86.3% (84.1–91.8)
Carbapenemases		
Sensitivity	95.5% (90.9–100)	100% (90.9–100)
Specificity	98.5% (98.5–98.5)	98.8% (98.8–98.8)

^*a*^ Three human experts (median).

^*b*^ Three independent prompting outputs from the customized GPT-4 agent “EUCAST-GPT-expert.” As reference standard, we used the results reported according to our ISO-accredited laboratory information system. ESBL, extended spectrum beta-lactamase; None, no specific molecular resistance mechanism.

Interestingly, when we focused on individual bacterial species, we observed a potential species-specific effect. As an example, in *E. coli* (*n* = 132), we noted lower ESBL detection rates compared to all samples (median sensitivity 86.4% vs 95.4%), and a lower median sensitivity compared to *K. pneumoniae* and *K. oxytoca* complex (*n* = 45, median sensitivity 100%, Table 2). However, the specificity in ESBL-producing *E. coli* was higher compared to all samples (median specificity 76.9% vs 69.2%), and higher compared to *K. pneumoniae* and *K. oxytoca* complex (median specificity 61.9%). This could potentially be explained by the previously mentioned misinterpretation of ESBL in the case of amoxicillin resistance and amoxicillin/clavulanic acid susceptibility.

**TABLE 2:** Comparison of common bacterial species and the performance of the EUCAST-GPT-expert^*a*^

	*Escherichia coli*	*Klebsiella pneumoniae* and *K. oxytoca*
ESBL		
Sensitivity	86.4% (83.7–92.7)	100% (100 - 100)
Specificity	76.9% (71.2–95.8)	61.9% (52.4–76.2)

^*a*^ Only ESBL was analyzed; as for AmpC- and carbapenemase-producing bacteria, the numbers were too low and not balanced. Outputs of *K. pneumoniae* and *K. oxytoca* were pooled to generate more robust numbers.

### Comparison of AI and human performance

Human experts generally exhibited higher specificity, particularly in the detection of ESBL and AmpC phenotypes, compared to the EUCAST-GPT-expert. However, the sensitivity was comparable between the human experts and the AI agent (Table 1). We also wanted to compare the performance of a customized GPT-agent with that of the non-customized version of GPT-4. Therefore, we also generated outputs using the same images and prompting strategy but with a non-customized GPT-4. In the non-customized GPT-4, only 169/862 (19.6%) categories could be interpreted. Of these, 137/169 (81.1%) categories agreed with routine diagnostics. For this subgroup, the available phenotypic categories were too low to provide robust enough calculations for sensitivities and specificities for individual resistance mechanisms.

### Analysis of argumentation

The EUCAST-GPT-expert’s argumentation was more detailed than replies from human experts, which could be beneficial for educational purposes or in complex cases where thorough explanations are warranted. However, this verbosity may not be practical in a routine diagnostic setting where brevity is preferred. The human experts used in median eight words (interquartile range [IQR] 4–11), indicating concise rationales for their decisions. In contrast, the EUCAST-GPT-expert provided more extensive explanations, with a median of 158 words (IQR 140–174) and suggested, in addition, also confirmation steps with a median of five words (IQR 4–9). Although we did not perform an in-depth analysis of the quality, we noted that the EUCAST-GPT-expert provided in some cases correct interpretations of, e.g., the phenotypic resistance category, but that the argumentation for the interpretation was not correct.

EUCAST-GPT-expert provided an additional text to specifically recommend a next confirmation step. In most situations, a correct follow-up assay was described, e.g., with a specific PCR, but occasionally, it was simply noted that a confirmatory assay is necessary.

The non-customized GPT-4 used in median 85 words (IQR 72–105) for reasoning and 0 words (IQR 0–0) to suggest confirmation steps. In very few situations, the subsequent confirmation steps were properly described.

### Human expert and EUCAST-GPT-expert concordance

The three human experts showed concordance in 814/862 (94.4%) total phenotypic categories. Concordance for ESBL, AmpC, and carbapenemases was 94.0%, 96.6%, and 98.6%, respectively. In contrast, three separated runs with the EUCAST-GPT-expert showed concordance in 706/862 (81.9%) total phenotypic categories. Concordance for ESBL, AmpC, and carbapenemase was 74.4%, 72.3%, and 97.7%, respectively.

## DISCUSSION

We highlight the potential and limitations of integrating a customized AI-agent, specifically GPT-4 and GPT-agents, to screen for suspected resistance mechanisms with Kirby–Bauer disk diffusion images. The GPT-agent’s comparable sensitivity to human experts in detecting ESBL, AmpC beta-lactamases, and carbapenemases is promising and underscores its ability to recognize resistance mechanisms effectively, a crucial aspect in combating AMR. Since EUCAST-GPT-expert was intended to be used as a pre-classification system to screen for potential resistance mechanisms, the focus is on sensitivity, and a lower specificity is therefore accepted. However, the GPT-agent’s tendency to over-flag, particularly AmpC in isolates with cefoxitin susceptibility and ESBL in isolates with amoxicillin resistance and amoxicillin/clavulanic acid susceptibility due to a narrow spectrum beta-lactamase, suggests areas for model refinement and calls for a careful approach during integration in clinical practice as it may result in critical delays as well as increased workloads and costs during confirmation. For carbapenemase detection, it seems that the customized AI agent performed slightly better than human experts; however, this may just be a coincidental finding, and more data would be needed for further investigations. To increase the specificity, additional adaptation of the prompt might be valuable, but as LLM versions change over time, the prompt might need to also be adapted over time. The detailed argumentation provided by the GPT-agent, while beneficial for educational purposes, may need to be streamlined for practical application in busy diagnostic labs. The customized-GPT-agent showed a clear benefit over a non-customized version.

The variability in interpretation among human experts illustrates the subjective nature of manual readings and the potential for AI to provide more standardized interpretations (8). On this occasion, there may also be different interpretations of AmpC, depending on whether only plasmid-mediated or both plasmid- and chromosome-mediated resistance is considered. Variability in plate reading and interpretation and, as a matter of fact, also human error needs to be considered in any diagnostic test report. However, the expertise and clinical judgment of human microbiologists remain invaluable, especially in complex or ambiguous cases. Also, different LLM may show variable results as indicated in a comparison of clinical microbiology scenarios between GPT-3.5 and GPT-4 (17). Importantly, GPT-4 and the customized-GPT agent do not provide detailed insights on what kind of data are entered initially in the data training set and how the data are analyzed and interpreted. In more open-source LLMs, the training data set could be added to the model. These systems remain a black box. Open-source LLMs, such as LLAMA-2, will become very important to explore the technology and understand how AI algorithms work with real-world data (9).

Our results demonstrate the importance of ongoing AI refinement, considering both the rapid evolution of AMR patterns and advancements in technology. The study also underscores the necessity of regulatory compliance and validation for AI tools in healthcare, as highlighted by the lack of IVDR/FDA approval for GPT-4 in clinical diagnostics (9). Thereby, our study may also be seen as a blueprint for a data set, which allows tracking of LLM progress and as a benchmark *in silico* data set. Repetitive testing of the same data set allows tracking performance evolution of LLMs.

Future studies should focus on expanding the data set to include a wider range of bacterial species and resistance mechanisms. Additionally, exploring AI’s role in interpreting other diagnostic tests could provide a more comprehensive understanding of its capabilities and limitations (11). Collaborations between AI developers, medical microbiologists, and regulatory bodies are essential to ensure that AI tools are safely and effectively integrated into clinical workflows.

Our study has important limitations. We have consciously opted for a commercial LLM that is accessible to a broad spectrum, but which is not further trainable by its user; therefore, the performance mostly depends on the pre-training of the specific version of GPT-4 used. As this field rapidly evolves, and releases are published on a regular basis, by the time this article is published, the performance has likely improved. Also, we have focused on Gram-negative bacteria and beta-lactamases. Our study was not powered to determine performance differences between humans and the customized GPT for individual resistance mechanisms. A sufficiently powered prospective validation will be necessary. Future studies should explore different bacterial species and resistance mechanisms, e.g., with methicillin-resistant *Staphylococcus aureus* or vancomycin- resistant *Enterococcus faecium*. Moreover, the AI’s performance in a real-world clinical setting may differ from this controlled study environment. There are many different combinations of carbapenemase and ESBL-producing strains—we cannot be sure that all combinations will work. A larger even more heterogenous data set may clarify some of the potential combinations. Prospective trials are needed in the field of AI and laboratory investigations; however, a first step must be a retrospective assessment to ensure its safety and baseline performance. Next, we have only included a few *Acinetobacter baumannii* and *Pseudomonas aeruginosa* isolates. Future work needs a more balanced data set. Finally, our data set has only been used in a GPT-4- and GPT-agent-related context and has not been used to explore other LLMs.

## Data Availability

The data can be accessed at the Dryad data repository under https://doi.org/10.5061/dryad.5dv41nsfj

## SUPPLEMENTAL MATERIAL

Figure S1 jcm.00689-24-s0001.jpg Design of the customized GPT.

Figure S2 jcm.00689-24-s0002.jpg Representative photography of a disk diffusion assay generated by the SIRscan device.

Figure S3 jcm.00689-24-s0003.jpg Representative photography of a disk diffusion assay generated by the SIRscan device.

Supplemental legends jcm.00689-24-s0004.docx Legends for supplemental tables and figures.

Table S1 jcm.00689-24-s0005.docx A representative isolate (isolate 2.3.1) with measured inhibition zone diameters.

Table S2 jcm.00689-24-s0006.docx Representative output table from the EUCAST-GPT-expert and the human expert.

ASM does not own the copyrights to Supplemental Material that may be linked to, or accessed through, an article. The authors have granted ASM a non-exclusive, world-wide license to publish the Supplemental Material files. Please contact the corresponding author directly for reuse.
