# Supplementary figures and images for: GPT-4-based AI agents—the new expert system for detection of antimicrobial resistance mechanisms?
Source: J Clin Microbiol. 2024 Oct 17;62(11):e00689-24. doi: 10.1128/jcm.00689-24 (PMC11559085; doi:10.1128/jcm.00689-24)

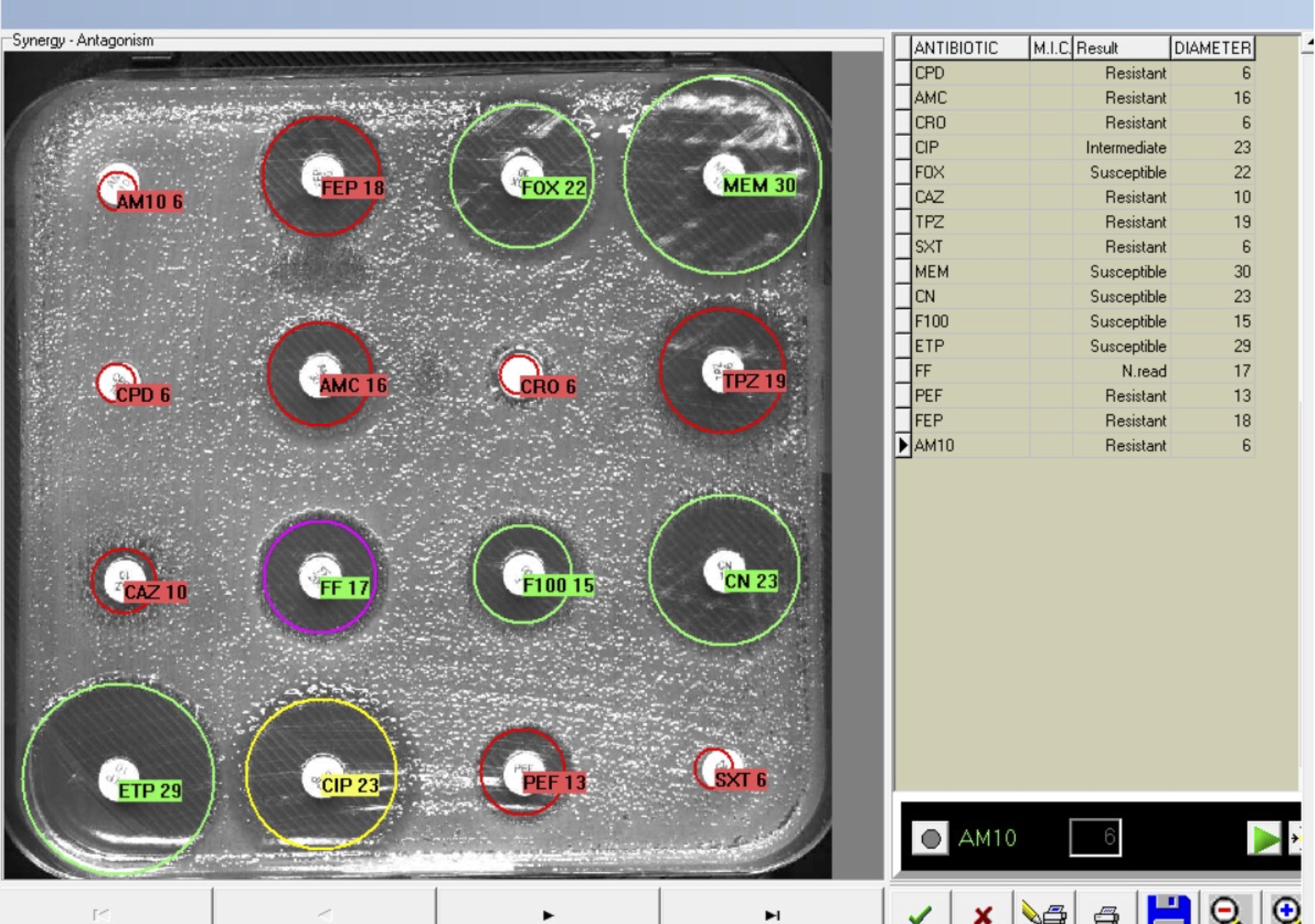

Supplement: Figure S2 — Representative photography of a disk diffusion assay generated by the SIRscan device. [file jcm.00689-24-s0002.jpg]

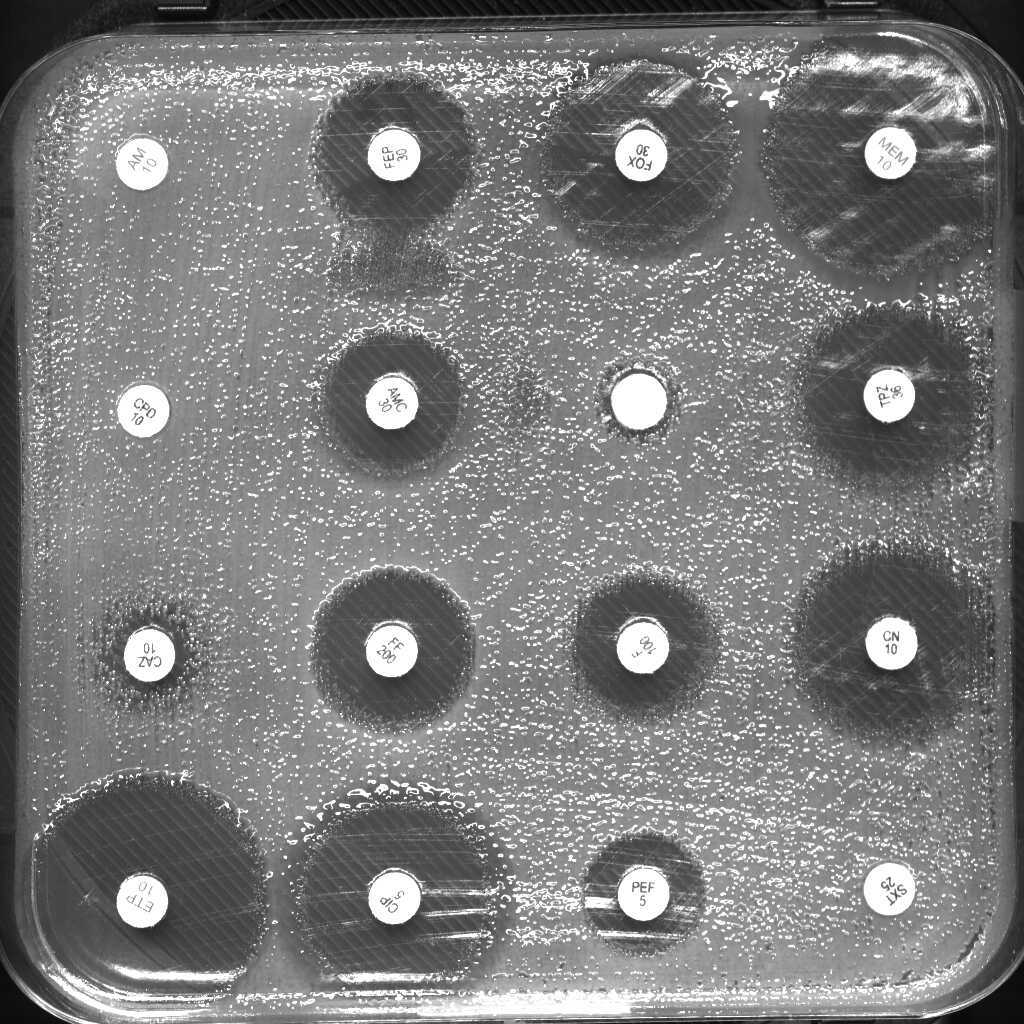

Supplement: Figure S3 — Representative photography of a disk diffusion assay generated by the SIRscan device. [file jcm.00689-24-s0003.jpg]
