# Supplementary material for: GPT-4-based AI agents—the new expert system for detection of antimicrobial resistance mechanisms?
Source: J Clin Microbiol. 2024 Oct 17;62(11):e00689-24. doi: 10.1128/jcm.00689-24 (PMC11559085; doi:10.1128/jcm.00689-24)
Supplement: Supplemental legends — Legends for supplemental tables and figures. [file jcm.00689-24-s0004.docx]

**Supplementary material.**

**Supplementary Figure 1. Design of the customized GPT.** The image shows the screenshot of the configuration tab of the generated customized bot with the browser-based software.

**Supplementary Figure 2. Representative photography of a disk diffusion assay generated by the SIRscan device.** The image originates from routine diagnostics and shows measured circles to indicate the inhibition zone diameters in mm and a categorical interpretation according to EUCAST. As a representative example image 2.3.1 was used.

**Supplementary Figure 3. Representative photography of a disk diffusion assay generated by the SIRscan device.** The image originates from routine diagnostics and shows no circles to indicate the measured inhibition zone diameters. As a representative example image 2.3.1 was used.

**Supplementary Table 1. A representative isolate (isolate 2.3.1) with measured inhibition zone diameters.** The measured inhibition zones were interpreted according to EUCAST. As a representative example image 2.3.1 was used. R, resistant; I, susceptible, increased exposure; S, susceptible; N. read, not read.

**Supplementary Table 2. Representative output table from the EUCAST-GPT-expert and the human expert.** In this example sample 6.70.1: *Escherichia coli* is shown.
