# Supplementary material for: GPT-4-based AI agents—the new expert system for detection of antimicrobial resistance mechanisms?
Source: J Clin Microbiol. 2024 Oct 17;62(11):e00689-24. doi: 10.1128/jcm.00689-24 (PMC11559085; doi:10.1128/jcm.00689-24)
Supplement: Table S1 — A representative isolate (isolate 2.3.1) with measured inhibition zone diameters. [file jcm.00689-24-s0005.docx]

| **Abbreviation** | **Antibiotic** | **Inhibition zone (mm)** | **Interpretation (S/I/R)** | **Resistant (mm)** | **Susceptible**  **(mm)** |
| --- | --- | --- | --- | --- | --- |
| CPD | Cefpodoxime | 6 | R | <21 | $\geq$21 |
| AMC | Amoxicillin/Clavulanic acid | 16 | R | <16 | $\geq$16 |
| CRO | Ceftriaxone | 6 | R | $<$22 | $\geq$25 |
| CIP | Ciprofloxacin | 23 | I | $<$22 | $\geq$25 |
| FOX | Cefoxitin | 22 | S | $<$19 | $\geq$19 |
| CAZ | Ceftazidime | 10 | R | $<$19 | $\geq$22 |
| TPZ | Piperacillin/Tazobactam | 19 | R | $<$20 | $\geq$20 |
| SXT | Sufamethoxazol-Trimethoprim | 6 | R | $<$11 | $\geq$14 |
| MEM | Meropenem | 30 | S | $<$16 | $\geq$22 |
| CN | Gentamicin (10ug) | 23 | S | $<$17 | $\geq$17 |
| F100 | Nitrofurantoin (100ug) | 15 | S | $<$11 | $\geq$11 |
| ETP | Ertapenem | 29 | S | $<$25 | $\geq$25 |
| FF | Fosfomycin | 17 | N. read | $<$24 | $\geq$24 |
| PEF | Pefloxacin | 13 | R | $<$24 | $\geq$24 |
| FEP | Cefepime | 18 | R | $<$24 | $\geq$27 |
| AM10 | Ampicillin (10ug) | 6 | R | $<$14 | $\geq$14 |

**Supplementary Table 1. A representative isolate (isolate 2.3.1) with measured inhibition zone diameters.** The measured inhibition zones were interpreted according to EUCAST. As representative example image 2.3.1 was used. R, resistant; I, susceptible, increased exposure; S, susceptible; N. read, not read.
