# Supplementary material for: GPT-4-based AI agents—the new expert system for detection of antimicrobial resistance mechanisms?
Source: J Clin Microbiol. 2024 Oct 17;62(11):e00689-24. doi: 10.1128/jcm.00689-24 (PMC11559085; doi:10.1128/jcm.00689-24)
Supplement: Table S2 — Representative output table from the EUCAST-GPT-expert and the human expert. [file jcm.00689-24-s0006.docx]

| **Isolate** | **EUCAST GPT Expert 1** | **EUCAST GPT Expert 2** | **EUCAST GPT Expert 3** | **Human Expert 1** | **Human Expert 2** | **Human Expert 3** |
| --- | --- | --- | --- | --- | --- | --- |
| *E. coli* with ESBL  (6.70.1.) | **None**: The presence of resistance to several beta-lactams, including cefpodoxime (CPD), and sensitivity to meropenem (MEM) suggests a resistance mechanism is present​​. **ESBL-production**: The resistance to cefpodoxime (CPD) and sensitivity to piperacillin/tazobactam (TPZ) align with an ESBL-producing phenotype, as ESBL producers are typically resistant to third-generation cephalosporins but may remain susceptible to beta-lactam/beta-lactamase inhibitor combinations​​. **AmpC-production**: Resistance to cefoxitin (FOX) is indicative of AmpC beta-lactamase production. This enzyme confers resistance to penicillins, cephamycins like cefoxitin, and often to the oxyimino-cephalosporins such as ceftriaxone (CRO)​​. **Carbapenemase production**: Susceptibility to ertapenem (ETP) and meropenem (MEM) indicates that carbapenemase production is very unlikely. Carbapenemase-producing organisms are typically resistant to carbapenems​​. For confirmation, phenotypic tests like ESBL Etests or genotypic methods such as PCR for ESBL genes can be used to confirm ESBL production. AmpC production could be investigated with a cefoxitin-cloxacillin double-disk synergy test (CC-DDS) or boronic acid-based tests if clinically indicated. Given the very unlikely presence of carbapenemase production indicated by the susceptibility pattern, no further tests are required for this mechanism. | **None:** The isolate is susceptible to meropenem (MEM), indicating that it is unlikely to have a carbapenemase resistance mechanism. It is also susceptible to ertapenem (ETP), further supporting the absence of carbapenemase production. However, resistance to several other antibiotics suggests the presence of another resistance mechanism. **ESBL-production:** The isolate is resistant to cefpodoxime (CPD), which suggests ESBL production as ESBL-producing organisms often show reduced susceptibility to third-generation cephalosporins. Additionally, resistance to ampicillin (AM10) and amoxicillin/clavulanic acid (AMC) further supports this hypothesis, as ESBL producers can hydrolyze penicillins and are often resistant to amoxicillin/clavulanic acid. It should be noted, however, that *E. coli* is intrinsically resistant to ampicillin, which alone does not indicate ESBL production. **AmpC-production:** The isolate is susceptible to cefoxitin (FOX), which argues against AmpC beta-lactamase production as these enzymes typically confer resistance to cefoxitin. **Carbapenemase production:** The pronounced susceptibility to meropenem (MEM) and ertapenem (ETP) suggests that carbapenemase production is very unlikely, as resistance or reduced susceptibility to carbapenems would be expected in the presence of carbapenemase enzymes. **Confirmation Tests:** Given the likelihood of ESBL production, it is recommended to perform confirmatory tests such as the double-disk synergy test (DDST) or combined disk test, particularly if clinical suspicion persists. No further confirmatory tests are recommended for AmpC or carbapenemase production due to the susceptibility patterns observed. | *None*: The pattern of susceptibility and resistance does not suggest a complete absence of resistance mechanisms. *ESBL-production*: Resistance to cefpodoxime (CPD 6), and intermediate resistance to cefepime (FEP 25) and ceftazidime (CAZ 15), while maintaining susceptibility to meropenem (MEM 30), is indicative of ESBL production. ESBL-producers often show this pattern of cephalosporin resistance while remaining susceptible to carbapenems. *AmpC-production*: The susceptibility to cefoxitin (FOX 23) suggests that AmpC production is unlikely, as EUCAST guidelines indicate that organisms producing AmpC beta-lactamase typically show resistance to cefoxitin. *Carbapenemase production*: The strong susceptibility to meropenem (MEM 30) indicates that carbapenemase production is very unlikely. Resistance to meropenem would be expected if carbapenemase production were present. The above analysis is grounded in the AST results, which show a susceptibility pattern typical of ESBL-producers according to EUCAST definitions. While the susceptibility to meropenem helps rule out carbapenemase production, the resistance and intermediate resistance patterns to cephalosporins suggest ESBL production, warranting confirmatory testing for clinical decision-making. | esbl present, weak synergy between AMC and FEP present | FOX-S/FEP-R and synergy AMC, true ESBL? inhibitor combinations with weak activity, not typical for ESBL | ESBL:CPD highly R, sinergy between AMC/CRO and AMC/FEP; AmpC: FOX S; Carba ETP S |
|  |  |  |  |  |  |  |
| *E. coli*  (1.1.1.) | **None**: The antibiogram indicates that the *E. coli* isolate is susceptible to all tested antibiotics, including broad-spectrum cephalosporins like cefpodoxime (CPD), ceftriaxone (CRO), and ceftazidime (CAZ), as well as the carbapenems meropenem (MEM) and ertapenem (ETP). This suggests that the isolate does not possess resistance mechanisms against these classes of antibiotics. **ESBL-production**: The susceptibility of the isolate to cefpodoxime (CPD 28 mm) and ceftazidime (CAZ 28 mm) suggests that ESBL production is unlikely, as ESBL-producing organisms typically show resistance to these third-generation cephalosporins. **AmpC-production**: The isolate is susceptible to cefoxitin (FOX 26 mm), which implies that AmpC production is unlikely because organisms producing AmpC β-lactamase usually exhibit resistance or decreased susceptibility to cefoxitin. **Carbapenemase production**: The isolate is susceptible to both meropenem (MEM 34 mm) and ertapenem (ETP 38 mm), which strongly suggests that carbapenemase production is very unlikely, as this mechanism leads to resistance to carbapenems. For confirmation, while it is unlikely that ESBL or AmpC production is present, if there is clinical suspicion, ESBL production can be further investigated using the double-disk synergy test (DDST) or combined disk test. AmpC production could be explored with a cefoxitin-cloxacillin double-disk synergy test (CC-DDS) if deemed necessary. Given the antibiogram results, no further testing for carbapenemase production is recommended. | **None**: The susceptibility to a broad range of antibiotics, including higher-generation cephalosporins like cefpodoxime (CPD), ceftazidime (CAZ), and carbapenems such as meropenem (MEM) and ertapenem (ETP), suggests the absence of ESBL, AmpC, or carbapenemase resistance mechanisms. **ESBL-production**: The organism shows susceptibility to cefpodoxime (CPD 28 mm) and ceftazidime (CAZ 28 mm), which would typically be resistant in ESBL-producing strains. The absence of resistance to amoxicillin-clavulanic acid (AMC 22 mm) further supports this. **AmpC-production**: The susceptibility to cefoxitin (FOX 26 mm) indicates that AmpC beta-lactamase production is unlikely since AmpC producers typically exhibit reduced susceptibility to cefoxitin. **Carbapenemase production**: Given the large zones of inhibition for meropenem (MEM 34 mm) and ertapenem (ETP 38 mm), carbapenemase production is very unlikely as organisms with this mechanism would show resistance or reduced susceptibility to these antibiotics. For confirmation, if clinically indicated despite the susceptibility pattern, ESBL production can be investigated using the double-disk synergy test (DDST) or combined disk test. AmpC production might be investigated with a cefoxitin-cloxacillin double-disk synergy test (CC-DDS) if necessary. However, given the very likely absence of these mechanisms as indicated by the susceptibility pattern, confirmation tests for carbapenemase production are not required. In the event that clinical data or patient history suggests a high risk of resistance not detected by this in vitro testing, molecular methods or sequencing could be considered to rule out resistance mechanisms. However, based on the available data, it is very likely that this *E. coli* isolate does not harbor any of the resistance mechanisms tested for. | **None**: The susceptibility to all tested antibiotics implies the absence of any detected resistance mechanism. **ESBL-production**: The susceptibility to third and fourth-generation cephalosporins makes ESBL production unlikely. **AmpC-production**: The susceptibility to Cefoxitin, which is resistant to degradation by AmpC beta-lactamases, suggests the absence of AmpC production. **Carbapenemase production**: The pronounced susceptibility to both Meropenem and Ertapenem makes carbapenemase production very unlikely. For confirmation, if clinically indicated, ESBL production could be further investigated using the double-disk synergy test (DDST) or a combined disk test. Given the very likely absence of AmpC and carbapenemase, no further tests are required for these mechanisms. | all beta lactam antimicrobials susceptible | all susceptible | Ampicillin S |
|  |  |  |  |  |  |  |
| *K. pneumoniae* NDM, ESBL  (7.11.1.) | **None**: No susceptibility to any tested antibiotics indicates the presence of multiple resistance mechanisms. **ESBL-production**: Resistance to third-generation cephalosporins such as ceftriaxone and ceftazidime, as well as to cefpodoxime, is a strong indication of ESBL production. **AmpC-production**: Susceptibility to cefoxitin would typically indicate the absence of AmpC, but resistance to cefoxitin in this case suggests the potential presence of AmpC production or other resistance mechanisms. **Carbapenemase production**: Resistance to ertapenem and meropenem is a strong indicator of carbapenemase production. Clinical correlation and additional testing with specific phenotypic or genotypic methods would be necessary to confirm these findings in a clinical setting. | **None**: The presence of resistance to all tested antibiotics, including broad-spectrum cephalosporins and carbapenems, indicates that there is a presence of a resistance mechanism **ESBL-production**: The resistance to third-generation cephalosporins like cefpodoxime (CPD) and ceftazidime (CAZ) suggests ESBL production as they typically confer reduced susceptibility to these agents. **AmpC-production**: Resistance to cefoxitin (FOX), a second-generation cephalosporin, indicates potential AmpC beta-lactamase production, which can confer resistance to penicillins and first-generation cephalosporins. **Carbapenemase production**: The resistance to ertapenem (ETP) and meropenem (MEM) suggests the possibility of carbapenemase production, as these enzymes typically reduce susceptibility to carbapenems. Please note that the presence of resistance to amoxicillin-clavulanic acid (AMC) alone does not necessarily indicate ESBL production due to *Klebsiella pneumoniae's* intrinsic resistance to amoxicillin. Additionally, the interpretation of these results must be cautious, and confirmatory testing should be performed to accurately determine the presence of these mechanisms. The laboratory should also check the quality of the susceptibility testing and species identification due to the unexpected resistance profile observed. | *None*: The resistance profile displayed, including resistance to broad-spectrum cephalosporins, indicates the presence of a resistance mechanism. *ESBL-production*: The resistance to third-generation cephalosporins such as cefotaxime (CPD) and ceftazidime (CAZ) alongside the resistance to amoxicillin/clavulanic acid (AMC) suggests ESBL production, which is often seen in *Klebsiella pneumoniae*. *AmpC-production*: The resistance pattern does not indicate AmpC production, as there is resistance to cefoxitin (FOX), which is not typically seen with AmpC producers. *Carbapenemase production*: The intermediate resistance to meropenem (MEM) suggests a possible reduced susceptibility to carbapenems, which could indicate carbapenemase production. This warrants further testing due to the clinical significance of this resistance mechanism. Please note that the recommendations are based on the EUCAST guidelines and the provided antibiogram data. The provided images do not show the zones of inhibition clearly, which is essential for accurate interpretation. Laboratory confirmation with phenotypic or genotypic methods is recommended to accurately determine the presence of these resistance mechanisms. | carbapenemase present, possible that underneathe ESBL also present | CPE very likely, confirmation required, combination of non-CPE mechanisms, however, possible | ESBL:FEP R-> CTX-MULTI LFIA or phenotypic confirmatory test to confirm ESBL production; AmpC: FOX and AMC highly R -> phenotypic confirmatory test required to confirm AmpC production; Carba:MEM R, Aminoglycoside high level, broad range resistance -> CM, TOB, AK (RMTase, usually associated withcarbapenemases) -> Carba-5 to confirm carbapenemase production and identify the type |
